# Supplementary material for: Ongoing resolution of duplicate gene functions shapes the diversification of a metabolic network
Source: eLife. 2016 Sep 30;5:e19027. doi: 10.7554/eLife.19027 (PMC5089864; doi:10.7554/eLife.19027)
Supplement: Figure 1—source data 1. — Quantitative data underlying Figure 1. DOI: http://dx.doi.org/10.7554/eLife.19027.004 [file elife-19027-fig1-data1.docx]

| *S. uvarum* | Identity to *S. cerevisiae* ortholog | Identity to other *S. cerevisiae* homolog | Identity to *S. uvarum* paralog | Functions of its *S. cerevisiae* ortholog |
| --- | --- | --- | --- | --- |
| Gal1 | 88.6% | 75.5% | 78.7% | Galactokinase |
| Gal2 | 89.9% | -- | 92.1% | Galactose transporter |
| Gal2b | 86.6% | -- | 92.1% | Galactose transporter |
| Gal3 | 83.3% | 76.6% | 78.7% | Co-inducer |
| Gal4 | 67.7% | -- | -- | Transcription factor |
| Gal7 | 83.9% | -- | -- | Galactose-1-phosphate uridyl transferase |
| Gal10 | 87.4% | -- | -- | UDP-glucose-4-epimerase |
| Gal80 | 93.6% | -- | 72.1% | Co-repressor |
| Gal80b | -- | 71.9% | 72.1% | -- |

Figure 1-Source data 1. Amino acid identity and *GAL* gene composition between *S. uvarum* and *S. cerevisiae GAL* network. Quantitative data underlying Figure 1.
