# Supplementary material for: Ongoing resolution of duplicate gene functions shapes the diversification of a metabolic network
Source: eLife. 2016 Sep 30;5:e19027. doi: 10.7554/eLife.19027 (PMC5089864; doi:10.7554/eLife.19027)
Supplement: Supplementary file 2. — DOI: http://dx.doi.org/10.7554/eLife.19027.024 [file elife-19027-supp2.docx]

Supplementary File 2. List of strains used in this study

| Strain number | Species | Genotype | Note |
| --- | --- | --- | --- |
| yHMK 56 | *S. cerevisiae* | *MAT***a** *ura3-∆ lys2-∆ chrIR::P_TDH3_-yEGFP-T_CYC1_* | Used as *S. cerevisiae* wild-type in each experiment; S288c derivative (Hittinger and Carroll 2007) |
| yHMK 853 | *S. cerevisiae* | *MAT***a** *ura3-∆ lys2-∆ chrIR::P_TDH3_-yEGFP-T_CYC1_ gal1∆::URA3* | Transformant derived from yHMK 56 (Hittinger and Carroll 2007) |
| yHMK 370 | *S. cerevisiae* | *MAT***a** *ura3-∆ lys2-∆ chrIR::P_TDH3_-yEGFP-T_CYC1_ gal1-atg1ctg* | Used as *S. cerevisiae gal1* mutant (Hittinger and Carroll 2007) |
| yHMK 371 | *S. cerevisiae* | *MAT***a** *ura3-∆ lys2-∆ chrIR::P_TDH3_-yEGFP-T_CYC1_ gal1-atg1ctg* | Used as *S. cerevisiae gal1* mutant (Hittinger and Carroll 2007) |
| yHMK 372 | *S. cerevisiae* | *MAT***a** *ura3-∆ lys2-∆ chrIR::P_TDH3_-yEGFP-T_CYC1_ gal1-atg1ctg* | Used as *S. cerevisiae gal1* mutant (Hittinger and Carroll 2007) |
| yHMK 980 | *S. cerevisiae* | *MAT***a** *ura3-∆ lys2-∆ chrIR::P_TDH3_-yEGFP-T_CYC1_ gal1∆::P_ScerGAL1_-SuvaGAL3* | Transformant derived from yHMK 853 |
| yHMK 65 | *S. uvarum* | *MAT***a** *hoΔ::NatMX* | Used as *S. uvarum* wild-type in each experiment, CBS7001 derivative (Scannell et al. 2011) |
| yHMK 837 | *S. uvarum* | *MAT***a** *hoΔ::NatMX gal1∆::TK-KanMX* | Transformant derived from yHMK 65 |
| yHMK 838 | *S. uvarum* | *MAT***a** *hoΔ::NatMX gal1∆::TK-KanMX* | Transformant derived from yHMK 65 |
| yHMK 839 | *S. uvarum* | *MAT***a** *hoΔ::NatMX gal1∆::TK-KanMX* | Transformant derived from yHMK 65 |
| yHMK 844 | *S. uvarum* | *MAT***a** *hoΔ::NatMX gal3∆::HphMX* | Transformant derived from yHMK 65 |
| yHMK 845 | *S. uvarum* | *MAT***a** *hoΔ::NatMX gal3∆::HphMX* | Transformant derived from yHMK 65 |
| yHMK 846 | *S. uvarum* | *MAT***a** *hoΔ::NatMX gal3∆::HphMX* | Transformant derived from yHMK 65 |
| yHMK 908 | *S. uvarum* | *MAT***a** *hoΔ::NatMX gal3∆::HphMX gal1∆::P_SuvaGAL1_-ScerGAL1* | Transformant derived from yHMK 876 |
| yHMK 909 | *S. uvarum* | *MAT***a** *hoΔ::NatMX gal3∆::HphMX gal1∆::P_SuvaGAL1_-ScerGAL1* | Transformant derived from yHMK 876 |
| yHMK 812 | *S. uvarum* | *MAT***a** *hoΔ::NatMX gal80b∆::KanMX gal1∆::P_ScerGAL1_-SuvaGAL1* | Transformant derived from yHMK 267 (*GAL1* from S288c) |
| yHMK 850 | *S. uvarum* | *MAT***a** *hoΔ::NatMX gal3∆::HphMX gal1∆::P_ScerGAL1_-SuvaGAL1* | Transformant derived from yHMK 812 |
| yHMK 870 | *S. uvarum* | *MAT***a** *hoΔ::NatMX gal3∆::HphMX gal1∆::P_ScerGAL1_-SuvaGAL1* | Transformant derived from yHMK 812 |
| yHMK 871 | *S. uvarum* | *MAT***a** *hoΔ::NatMX gal3∆::HphMX gal1∆::P_ScerGAL1_-SuvaGAL1* | Transformant derived from yHMK 812 |
| yHMK 373 | *S. cerevisiae* | *MAT***a** *ura3-∆ lys2-∆ chrIR::P_TDH3_-yEGFP-T_CYC1_ gal3-atg1ctg* | Used as *S. cerevisiae gal3* mutant (Hittinger and Carroll 2007) |
| yHMK 374 | *S. cerevisiae* | *MAT***a** *ura3-∆ lys2-∆ chrIR::P_TDH3_-yEGFP-T_CYC1_ gal3-atg1ctg* | Used as *S. cerevisiae gal3* mutant (Hittinger and Carroll 2007) |
| yHMK 375 | *S. cerevisiae* | *MAT***a** *ura3-∆ lys2-∆ chrIR::P_TDH3_-yEGFP-T_CYC1_ gal3-atg1ctg* | Used as *S. cerevisiae gal3* mutant (Hittinger and Carroll 2007) |
| yHMK 266 | *S. uvarum* | *MAT***a** *hoΔ::NatMX gal80b∆::KanMX* | Transformant derived from yHMK 65 |
| yHMK 267 | *S. uvarum* | *MAT***a** *hoΔ::NatMX gal80b∆::KanMX* | Transformant derived from yHMK 65 |
| yHMK 268 | *S. uvarum* | *MAT***a** *hoΔ::NatMX gal80b∆::KanMX* | Transformant derived from yHMK 65 |
| yHMK 276 | *S. uvarum* | *MAT***a** *hoΔ::NatMX gal80∆::KanMX* | Transformant derived from yHMK 65 |
| yHMK 277 | *S. uvarum* | *MAT***a** *hoΔ::NatMX gal80∆::KanMX* | Transformant derived from yHMK 65 |
| yHMK 279 | *S. uvarum* | *MAT***a** *hoΔ::NatMX gal80∆::KanMX* | Transformant derived from yHMK 65 |
| yHMK 583 | *S. uvarum* | *MAT***a** *hoΔ::NatMX gal80∆::KanMX ψgto1∆::HphMX-P_GAL1_-EGFP-T_CYC1_* | Transformant derived from yHMK 276 |
| yHMK 784 | *S. uvarum* | *MAT***a** *hoΔ::NatMX gal80∆::KanMX ψgto1∆::HphMX-P_GAL1_-EGFP-T_CYC1_* | Transformant derived from yHMK 277 |
| yHMK 785 | *S. uvarum* | *MAT***a** *hoΔ::NatMX gal80∆::KanMX ψgto1∆::HphMX-P_GAL1_-EGFP-T_CYC1_* | Transformant derived from yHMK 277 |
| yHMK 581 | *S. uvarum* | *MAT***a** *hoΔ::NatMX gal80b∆::KanMX ψgto1∆::HphMX-P_GAL1_-EGFP-T_CYC1_* | Transformant derived from yHMK 266 |
| yHMK 582 | *S. uvarum* | *MAT***a** *hoΔ::NatMX gal80b∆::KanMX ψgto1∆::HphMX-P_GAL1_-EGFP-T_CYC1_* | Transformant derived from yHMK 266 |
| yHMK 584 | *S. uvarum* | *MAT***a** *hoΔ::NatMX gal80b∆::KanMX ψgto1∆::HphMX-P_GAL1_-EGFP-T_CYC1_* | Transformant derived from yHMK 266 |
| yHMK 280 | *S. uvarum* | *MAT***a** *hoΔ::NatMX gal80∆::TK-HphMX gal80b∆::KanMX* | Transformant derived from yHMK 267 |
| yHMK 281 | *S. uvarum* | *MAT***a** *hoΔ::NatMX gal80∆::TK-HphMX gal80b∆::KanMX* | Transformant derived from yHMK 267 |
| yHMK 282 | *S. uvarum* | *MAT***a** *hoΔ::NatMX gal80∆::TK-HphMX gal80b∆::KanMX* | Transformant derived from yHMK 267 |
| yHMK 405 | *S. uvarum* | *MAT***a** *hoΔ::NatMX gal80-∆ gal80b∆::KanMX* | Transformant derived from yHMK 281 |
| yHMK 406 | *S. uvarum* | *MAT***a** *hoΔ::NatMX gal80-∆ gal80b∆::KanMX* | Transformant derived from yHMK 281 |
| yHMK 407 | *S. uvarum* | *MAT***a** *hoΔ::NatMX gal80-∆ gal80b∆::KanMX* | Transformant derived from yHMK 281 |
| yHMK 593 | *S. uvarum* | *MAT***a** *hoΔ::NatMX gal80-∆ gal80b∆::KanMX ψgto1∆::HphMX-P_GAL1_-EGFP-T_CYC1_* | Transformant derived from yHMK 405 |
| yHMK 789 | *S. uvarum* | *MAT***a** *hoΔ::NatMX gal80-∆ gal80b∆::KanMX ψgto1∆::HphMX-P_GAL1_-EGFP-T_CYC1_* | Transformant derived from yHMK 405 |
| yHMK 790 | *S. uvarum* | *MAT***a** *hoΔ::NatMX gal80-∆ gal80b∆::KanMX ψgto1∆::HphMX-P_GAL1_-EGFP-T_CYC1_* | Transformant derived from yHMK 405 |
| yHMK 70 | *S. cerevisiae* | *MAT***a** *leu2∆ ura3∆ ho::KanMX* | RM11-1a (Brem et al. 2002) |
| yHMK 346 | *S. cerevisiae* | *MAT***a** *leu2∆ ura3∆ ho::KanMX gal80∆::TK-HphMX* | Transformant derived from yHMK 70 |
| yHMK 348 | *S. cerevisiae* | *MAT***a** *leu2∆ ura3∆ ho::KanMX gal80∆::TK-HphMX* | Transformant derived from yHMK 70 |
| yHMK 682 | *S. uvarum* | *MAT***a** *hoΔ::NatMX gal80-∆ gal80b∆::KanMX gal1∆::P_SuvaGAL1_-ScerGAL1* | Transformant derived from yHMK 624 (*GAL1* from S288c) |
| yHMK 696 | *S. uvarum* | *MAT***a** *hoΔ::NatMX gal80-∆ gal80b∆::KanMX gal1∆::P_SuvaGAL1_-ScerGAL1* | Transformant derived from yHMK 625 (*GAL1* from S288c) |
| yHMK 874 | *S. uvarum* | *MAT***a** *hoΔ::NatMX gal80b∆::KanMX gal80∆::HphMX gal1∆::P_ScerPGAL1_-SuvaGAL1* | Transformant derived from yHMK 812 (*GAL1* from S288c) |
| yHMK 876 | *S. uvarum* | *MAT***a** *hoΔ::NatMX gal1∆::P_SuvaPGAL1_-ScerGAL1* | Transformant derived from yHMK 838 (*GAL1* from RM11-1a) |
| yHMK 877 | *S. uvarum* | *MAT***a** *hoΔ::NatMX gal1∆::P_SuvaPGAL1_-ScerGAL1* | Transformant derived from yHMK 838 (*GAL1* from RM11-1a) |
| yHMK 878 | *S. uvarum* | *MAT***a** *hoΔ::NatMX gal1∆::P_SuvaPGAL1_-ScerGAL1* | Transformant derived from yHMK 838 (*GAL1* from RM11-1a) |
| yHMK 861 | *S. uvarum* | *MAT***a** *hoΔ::NatMX gal1∆::P_ScerPGAL1_-SuvaGAL1* | Transformant derived from yHMK 834 (*GAL1* from S288c) |
| yHMK 862 | *S. uvarum* | *MAT***a** *hoΔ::NatMX gal1∆::P_ScerPGAL1_-SuvaGAL1* | Transformant derived from yHMK 832 (*GAL1* from S288c) |
| yHMK 863 | *S. uvarum* | *MAT***a** *hoΔ::NatMX gal1∆::P_ScerPGAL1_-SuvaGAL1* | Transformant derived from yHMK 834 (*GAL1* from S288c) |
| yHMK 624 | *S. uvarum* | *MAT***a** *hoΔ::NatMX gal80-∆ gal80b∆::KanMX gal1∆::TK-HphMX* | Transformant derived from yHMK 405 |
| yHMK 625 | *S. uvarum* | *MAT***a** *hoΔ::NatMX gal80-∆ gal80b∆::KanMX gal1∆::TK-HphMX* | Transformant derived from yHMK 405 |
| yHMK 626 | *S. uvarum* | *MAT***a** *hoΔ::NatMX gal80-∆ gal80b∆::KanMX gal1∆::TK-HphMX* | Transformant derived from yHMK 406 |
| yHCT 322 | *S. cerevisiae* | *MAT***a** *ura3-∆ lys2-∆ chrIR::P_TDH3_-yEGFP-T_CYC1_ gal7∆::URA3* | Transformant derived from yHMK 56 |
| yHCT 324 | *S. cerevisiae* | *MAT***a** *ura3-∆ lys2-∆ chrIR::P_TDH3_-yEGFP-T_CYC1_ gal10∆::URA3* | Transformant derived from yHMK 56 |
| yHMK 457 | *S. uvarum* | *MAT***a** *hoΔ::NatMX gal80∆::KanMX gal80b∆::TK-HphMX* | Transformant derived from yHMK 276 |
| yHMK 458 | *S. uvarum* | *MAT***a** *hoΔ::NatMX gal80∆::KanMX gal80b∆::TK-HphMX* | Transformant derived from yHMK 276 |
| yHMK 459 | *S. uvarum* | *MAT***a** *hoΔ::NatMX gal80∆::KanMX gal80b∆::TK-HphMX* | Transformant derived from yHMK 276 |
| yHMK 356 | *S. uvarum* | *MAT***a** *hoΔ::NatMX gal80∆::P_SuvaGAL80_-ScerGAL80 gal80b∆::KanMX* | Transformant derived from yHMK 281 |
| yHMK 360 | *S. uvarum* | *MAT***a** *hoΔ::NatMX gal80b∆::KanMX gal80∆::P_SuvaGAL80_-SuvaGAL80* | Transformant derived from yHMK 281 |
| yHMK 361 | *S. uvarum* | *MAT***a** *hoΔ::NatMX gal80b∆::KanMX gal80∆::P_SuvaGAL80_-SuvaGAL80* | Transformant derived from yHMK 281 |
| yHMK 362 | *S. uvarum* | *MAT***a** *hoΔ::NatMX gal80b∆::KanMX gal80∆::P_SuvaGAL80_-SuvaGAL80* | Transformant derived from yHMK 281 |
| yHMK 925 | *S. cerevisiae* | *MAT***a** *ura3-∆ lys2-∆ chrIR::P_TDH3_-yEGFP-T_CYC1_ gal1∆::TK-HphMX* | Transformant derived from yHMK 56 |
| yHMK 978 | *S. cerevisiae* | *MAT***a** *ura3-∆ lys2-∆ chrIR::P_TDH3_-yEGFP-T_CYC1_ gal1∆::P_SuvaGAL1_-ScerGAL1* | Transformant derived from yHMK 925 |
